# Design, Synthesis, Evaluation and Thermodynamics of 1-Substituted Pyridylimidazo[1,5-*a*]Pyridine Derivatives as Cysteine Protease Inhibitors

**DOI:** 10.1371/journal.pone.0069982

**Published:** 2013-08-05

**Authors:** Mohd Sajid Khan, Mohd Hassan Baig, Saheem Ahmad, Shapi Ahmad Siddiqui, Ashwini Kumar Srivastava, Kumar Venkatraman Srinivasan, Irfan A. Ansari

**Affiliations:** 1 Division of Biochemical Science, National Chemical Laboratory, Pune, India; 2 Department of Biotechnology, Integral University, Dasauli, Lucknow, India; 3 Organic Chemistry Technology Division, National Chemical Laboratory, Pune, India; Aligarh Muslim University, India

## Abstract

Targeting papain family cysteine proteases is one of the novel strategies in the development of chemotherapy for a number of diseases. Novel cysteine protease inhibitors derived from 1-pyridylimidazo[1,5-*a*]pyridine representing pharmacologically important class of compounds are being reported here for the first time. The derivatives were initially designed and screened *in silico* by molecular docking studies against papain to explore the possible mode of action. The molecular interaction between the compounds and cysteine protease (papain) was found to be very similar to the interactions observed with the respective epoxide inhibitor (E-64c) of papain. Subsequently, compounds were synthesized to validate their efficacy in wet lab experiments. When characterized kinetically, these compounds show their K_i_ and IC_50_ values in the range of 13.75 to 99.30 µM and 13.40 to 96.50 µM, respectively. The thermodynamics studies suggest their binding with papain hydrophobically and entropically driven. These inhibitors also inhibit the growth of clinically important different types of Gram positive and Gram negative bacteria having MIC_50_ values in the range of 0.6–1.4 µg/ml. Based on Lipinski’s rule of Five, we also propose these compounds as potent antibacterial prodrugs. The most active antibacterial compound was found to be 1-(2-pyridyl)-3-(2-hydroxyphenyl)imidazo[1,5-*a*]pyridine (**3a**).

## Introduction

Cysteine-protease inhibitors (CPI) have gained considerable attention over the last couple of decades and many classes of compounds are currently in human clinical trials for a number of diseases. Interest in papain family cysteine proteases as chemotherapeutic targets is derived from the recognition that they are critical to the life cycle or pathogenicity of many microorganisms. The cysteine proteases from *Streptococcus sp.* (streptopain) [1], *Staphylococcus sp.* (staphopain) [2], *Plasmodium falciparum* (falcipain-1, -2, and -3) and *Trypanosoma cruzi* (cruzipain) [3] are some of the most widely studied members of papain family which have been reported to be linked with severity of infection and various pathological conditions caused by these microorganisms.

The activation of the kallikrein-kinin pathway, which could be activated by more than sixteen bacterial proteases, is a mechanism that some pathogens exploit to ensure that there is a supply of nutrients to the site of infection by increasing vascular permeability. This has been shown to occur in infections with several microbial species, including *Pseudomonas, Serratia, Clostridium, Candida, Bacteroides, Porphyromonas* and *Staphylococcus sp.* [4]. Many bacteria secrete several nonspecific proteases e.g. *Pseudomonas, Serratia, Streptococcus, Staphylococcus* and *Bacteroides sp.* have potent metallo-, cysteine and serine proteases with broad ranges of activities [5]. The critical role of bacterial proteases in virulence was successfully demonstrated by eliminating the protease-encoding gene in *P. gingivalis* [6].

Recently described cystatin superfamily of proteins comprises both eukaryotic and prokaryotic cysteine protease inhibitors [7]. Human cystatins C, D and S, rat cystatins A and S, chicken cystatin and oryza cystatin have been reported to inhibit the replication of certain viruses and bacteria [8] although it has not yet been directly demonstrated that these effects are due to the protease inhibitory capacity of the cystatins [9]. The key role of cysteine proteases in microbial infections, coupled with the relative lack of redundancy compared to mammalian systems has made microbial proteases attractive targets for the development of novel chemotherapeutic approaches [10], [11].

Imidazopyridine ring systems represent an important class of compounds not only for their theoretical interest but also from a pharmacological point of view. They have been shown to possess a broad range of useful pharmacological activities [12] including antigastric, antisecretory, local anesthetic, antiviral, antianxiety, antibacterial, antifungal, antihelminthic, antiprotozoal, anticonvulsant, gastrointestinal, antiulcer (Zolmidine), anxiolytic (Alpidem), hypnotic (Zolpidem) and immunomodulatory [13]. The nature and the position of the substituents on the pyridinic moiety influence these pharmacological activities. These imidazopyridine heterocyclic structures form part of the skeleton of natural alkaloids, neuromuscular blocking agents [14], reversible inhibitors of the H^+^, K^+^-ATPase enzymes with a potent antisecretory activity, and are known to be sedative hypnotics of the nervous system [15]. In this study, we have proposed kinetically and thermodynamically characterized 1-substituted pyridylimidazo[1,5-*a*]pyridine derivatives as a potent and novel cysteine protease inhibitors which also acts as antibacterial agents.

## Materials and Methods

### Materials

1-pyridylimidazo[1,5-a]pyridine; Microbes Staphylococcus aureus (ATCC-25923; Proteus vulgaris (ATCC-29905); Group D Streptococci (ATCC-12959); Bacillus spp. (ATCC-14593); Escherichia coli (ATCC-25922); Klebsiella pneumonia (ATCC-13883); Pseudomonas aeruginosa (ATCC-27853); Serratia morganii (ATCC- 31665) were obtained from Goa Medical College, Goa, India. Papain (EC 3.4.22.2) and Bz-DL-Arg-pNA (BAPNA) were purchased from Sigma Chemical Co., St. Louis, MO, USA. All solvents and chemicals were of analytical grade.

### Computational Studies: Preparation of Enzyme and Compounds for Docking

The crystal structure of papain was extracted from Protein Data Bank (PDB code: 1PE6) [16]. All the water molecules and heteroatoms were removed and hydrogen atoms were added to the protein. CharMm forcefield [17] was applied and the structure was subjected to energy minimization for 1000 steps using steepest descent method. The chemical structures of all the synthesized compounds were generated using chemdraw and were subsequently converted into 3D format using CORINA. A series of docking experiments were carried out with all the designed 1-substituted pyridylimidazo[1,5-*a*]pyridine derivatives against papain using AutoDock Tools 4.0 [18] for possible cysteine-protease inhibitory activities. The compounds were selected on the basis of their binding energies and those reflecting good binding affinity were further analyzed on *in silico* platform. As a parameter for the molecular docking, the Lamarckian genetic algorithm, a combination between the genetic algorithm and the local search Pseudo-Solis and Wets algorithm, was employed. A grid box of 60×60×60 Å was generated around active site of papain making sure those inhibitors can freely rotate inside the grid. The number of docking runs was set to 10. Each docking was repeated five times, having in the end a total of 50 docking runs, to check the precision of results. The finally obtained docked complexes were subsequently visualized using PyMol [19]. The work was further authenticated in the wet lab after its detailed analysis on *in silico* platform.

### Drug Likeness and *in silico* Toxicity Studies

The designed derivatives were filtered by Lipinski’s “Rule of five” that sets the criteria for drug-like properties. Drug likeness is a property that is most often used to characterize novel lead compounds [20]. According to this rule, poor absorption is expected if MW >500, log P>5, hydrogen bond donors >5, and hydrogen bond acceptors >10 [21]. In silico absorption, distribution, metabolism and excretion (ADME) properties of these derivatives were also predicted using following online bioinformatics tools.

- http://www.organic-chemistry.org.
- http://mobyle.rpbs.univ-paris-diderot.fr/cgi-bin/portal. py? Form = admetox
- https://secure.chemsilico.com/pages/submit.php

The above study gave us an idea about the existence of possible mutagenic and tumorigenic properties in synthesized compounds. The result obtained helped us to screen out the synthesized compounds for their further usage as potent leads.

### Synthesis of 1-substituted Pyridylimidazo[1,5-*a*]pyridine Derivatives

**Figure 1:**
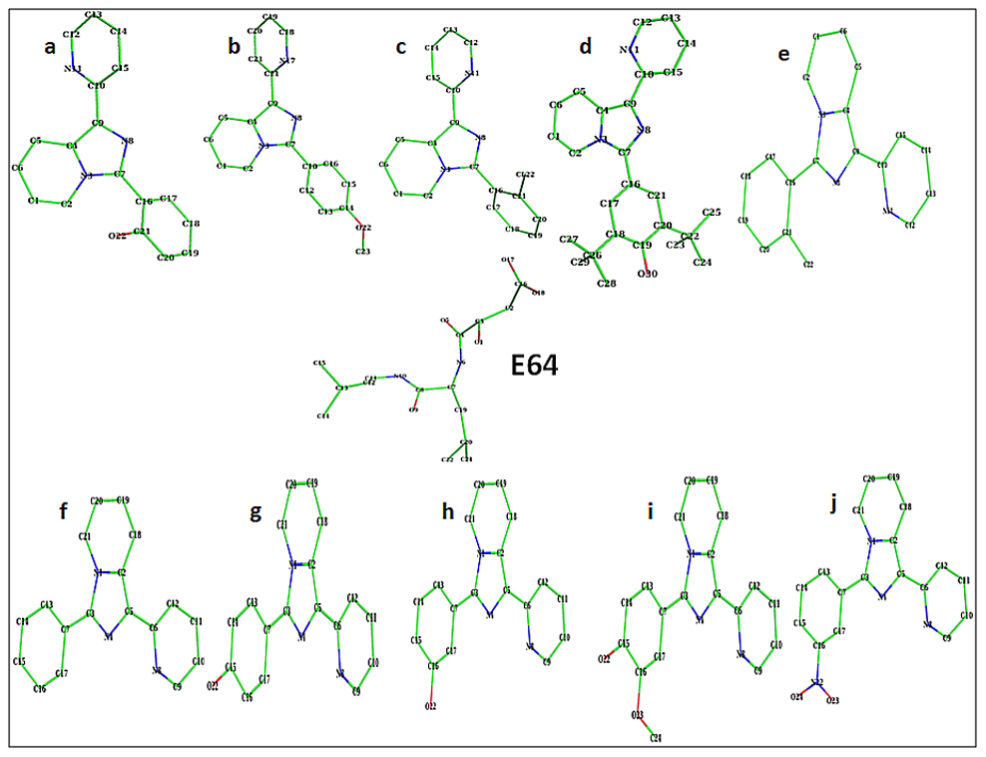
Chemical structures of 1–substituted pyridylimidazo[1,5-*a*]pyridine derivatives (3a–j).

Based on the results of docking studies, ten derivatives of 1-pyridylimidazo[1,5-a]pyridine were synthesized according to Siddiqui *et al*., 2006 [22] which are named as follows: 1-(2-pyridyl)-3-(2-hydroxyphenyl) imidazo[1,5-*a*]pyridine **(3a);** 1-(2-pyridyl)-3-(4-methoxyphenyl) imidazo[1,5-*a*]pyridine **(3b);** 1-(2-pyridyl)-3-(2-chlorophenyl) imidazo[1,5-*a*]pyridine **(3c);** 1-(2-pyridyl)-3-(3,5-di-*tert*-butyl-4-hydroxyphenyl) imidazo[1,5-*a*]pyridine **(3d);** 1-(2-Pyridyl)-3-(2-methylphenyl)imidazo[1,5-*a*]pyridine **(3e);** 1-(2-Pyridyl)-3-phenylimidazo[1,5-*a*]pyridine **(3f);** 1-(2-Pyridyl)-3-(4-hydroxyphenyl)imidazo[1,5-*a*]pyridine **(3g);** 1-(2-Pyridyl)-3-(3-hydroxyphenyl)imidazo[1,5-*a*]pyridine **(3h);** 1-(2-Pyridyl)-3-(3-methoxy-4-hydroxyphenyl)imidazo[1,5-*a*]pyridine **(3i)** and 1-(2-Pyridyl)-3-(3-nitrophenyl)imidazo[1,5-*a*]pyridine **(3j)** (Figure 1).

### Assay for Cysteine Protease Inhibitory Activity of 1-substituted Pyridylimidazo[1,5-*a*] Pyridine Derivatives

The capacity of the 1-pyridylimidazo[1,5-*a*]pyridine derivatives to inhibit cysteine proteases was tested using papain as the model enzyme. The proteolytic activity of the reaction mixtures was determined using Bz-DL-Arg-pNA as the chromogenic substrate [23]. To solutions of active papain (final concentration: 0.05 mM) were added concentrated solutions of the different derivatives to final concentrations of 0.2 mM. After incubation for 30 min at 37°C, the substrate solution was added and after a further incubation for 20 min the reaction was stopped by the addition of 5% trichloric acid (TCA) acidified with 2.25% HCl and the absorbance of the reaction mixture was determined at a wavelength of 410 nm by Microplate Manager 4.0 (Bio-Rad laboratories). The same procedure was used at 32°C and 42°C for thermodynamics studies. The kinetic parameters for the substrate hydrolysis were determined by measuring the initial rate of enzymatic activity. The inhibition constant K_i_ was determined by Dixon method [24] and also by the Lineweaver–Burk equation. The K_m_ value was calculated from the double-reciprocal equation by fitting the data into the computer software Origin 6.1. The Lineweaver–Burk plot was used to determine the types of inhibition. For the kinetic analysis and rate constant determinations, the assays were carried out in triplicate, and the average value was considered throughout this work.

### Thermodynamics Analysis of 1-substituted Pyridylimidazo[1,5-*a*] Pyridine Derivatives

Free energy changes of inhibition of papain against 1- substituted pyridylimidazo[1,5-*a*] pyridine derivatives (ΔG) were determined by the equation,
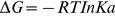
(1)


Temperature dependence of the inhibition constants was used to determine the thermodynamic parameters. Changes in enthalpy (ΔH) were determined from the Van’t Hoff plots by using the equation,
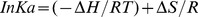
(2)


Where Δ*H* is enthalpy change, R is gas constant, ΔS is entropy change and T is the absolute temperature. The entropy change was obtained from the equation,
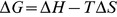
(3)


The assay was done at different temperatures (32°C, 37°C, 42°C) calculating various K_i_ of 1-pyridylimidazo[1,5-*a*]pyridine derivatives with papain as model enzyme.

### Determination of Minimum Inhibitory Concentration (MIC) of 1-substituted Pyridylimidazo[1,5-*a*] Pyridine Derivatives

The disk diffusion method [25] was used for the preliminary antibacterial evaluation of 1-pyridylimidazo[1,5-*a*]pyridine derivatives. The MIC_50_ of these derivatives, showing inhibition in the preliminary tests, were further determined by the microtitre plate technique using micro dilution method [26]. In brief, the bacterial strains (*S. aureus, P. vulgaris, Group D Streptococci*, *Bacillus sp.*, *E. coli*, *P. aeruginosa* and *S. morganii*)) were grown and diluted to 2×10^5^ colony-forming units (CFU)/ml in sodium phosphate buffer (SPB) containing 0.03% Luria–Bertani (LB) broth. The synthesized derivatives were dissolved in DMSO and their serial dilution was performed in 50 µL of LB medium in 96-well microtitre plate to achieve the required concentrations (0.1–10 µg/ml) with bacterial inoculums (5×10^4^ CFU per well). DMSO was taken as negative control and Ceftriaxone and clotrimazole were taken as positive control. After incubation at 37°C overnight, the MICs were taken as the lowest inhibitor concentration at which the bacterial growth was inhibited. The average of three values was calculated and that was the MIC for the test material and bacterial strain.

For the agar plate count method [27], 25 µL aliquots of bacteria at 1×10^5^ CFU/ml in SPB containing 0.03% LB broth were incubated with 25 µL of diluted compounds for 2 h at 37°C. The mixtures of bacteria and compounds were serially diluted 10-fold with SPB and plated on LB plates that were incubated at 37°C overnight. Bacterial colonies were enumerated the following day.

### Determination of Minimum Bactericidal Concentration (MBC) of 1-substituted Pyridylimidazo[1,5-*a*] Pyridine Derivatives

After having determined the MICs, bacterial strains from the wells of the microtitre plate with no visible bacterial growth were removed for serial sub cultivation of 2 µl into another new microtitre plate containing 100 µl of broth per well and further incubated for 24 h. The lowest concentration with no visible growth was defined as MBC [28], indicating 99.5% killing of the original inoculum. The absorbance of each well was measured at a wavelength of 620 nm by Microplate Manager 4.0 (Bio-Rad laboratories) and compared with a blank. Solvent (DMSO) was used as a negative control. Three replicates were done for each compound and experiment was repeated two times.

## Results and Discussion

Bacteria use their cysteine proteases for pathogenecity as could be depicted from the structure of Cif homolog in *Burkholderia pseudomallei* (CHBP) which reveals a papain-like fold and a conserved Cys-His-Gln catalytic triad [29]. It has been proven that bacterial pathogens have a unique papain-like hydrolytic activity to block the normal host cell cycle progression as the core of an avirulence (Avr) protein (AvrPphB) from the plant pathogen *Pseudomonas syringae,* resembles the papain-like cysteine proteases. The similarity of this AvrPphB protein with papain includes the catalytic triad of Cys-98, His-212, and Asp-227 in the AvrPphB active site [30].

Turk *et al.* have proposed, on the basis of kinetic and structural studies, that papain has seven subsites at the active site but only five subsites are important which can bind to an amino acid residue of the substrate [31]. A variety of intermediates are generated when papain reacts with substrate or an inhibitor [2]. Like serine proteases, cysteine proteases tend to have relatively shallow, solvent-exposed active sites that can accommodate short substrate/inhibitor segments of protein loops (e.g. from endogenous inhibitors such as cystatins) or strands. The inhibitor compound bound to protease with a combination of hydrogen bonds and hydrophobic interactions.

As a part of our investigation in developing novel and efficient cysteine protease inhibitors, ten 1-substituted pyridylimidazo[1,5-*a*]pyridine derivatives (**3a–j**) were primarily designed and screened on the basis of their docking energies against papain to elucidate their possible mode of action. It was found that these compounds were specific inhibitors of cysteine protease, papain and didn’t show inhibition against other types of proteases like serine, aspartic or metalloproteases. They are specific for CA clan of cysteine protease and didn’t show any significant inhibition against other clans of cysteine proteases.

These new compounds were devised based on the knowledge of ability of a protein to alter its conformation to accommodate a binding ligand and enabled us to directly compare the relative positions of the residue in the binding pocket. Molecular docking study provided the structural insight into the binding of these compounds (**3a–j**) **(** **Figure 1** **)** within the active site of papain which mainly consist of a catalytic triad of Cys 25, His 159 and Asp 175 [32]. Moreover, role of other residues present in the active site of papain, playing important role in the accommodation of compounds have also been revealed. Initially, docking was performed with all the designed compounds (**3a–j**) against papain, a known cysteine protease enzyme and in this context, we observed very interesting results where our proposed inhibitors (**3a–j**) take advantage of aromatic and hydrophilic residues by making a variety of interactions with target enzyme. Although, compounds **3e–j** gave significant results when docked with papain but during evaluation of antibacterial properties in wet lab experiments, they gave insignificant results (data not shown). Therefore, only four compounds were considered for discussion and further experiments like kinetic and thermodynamic studies to characterize these compounds as potent pro-inhibitors, were performed (**3a–d**).

**Figure 2:**
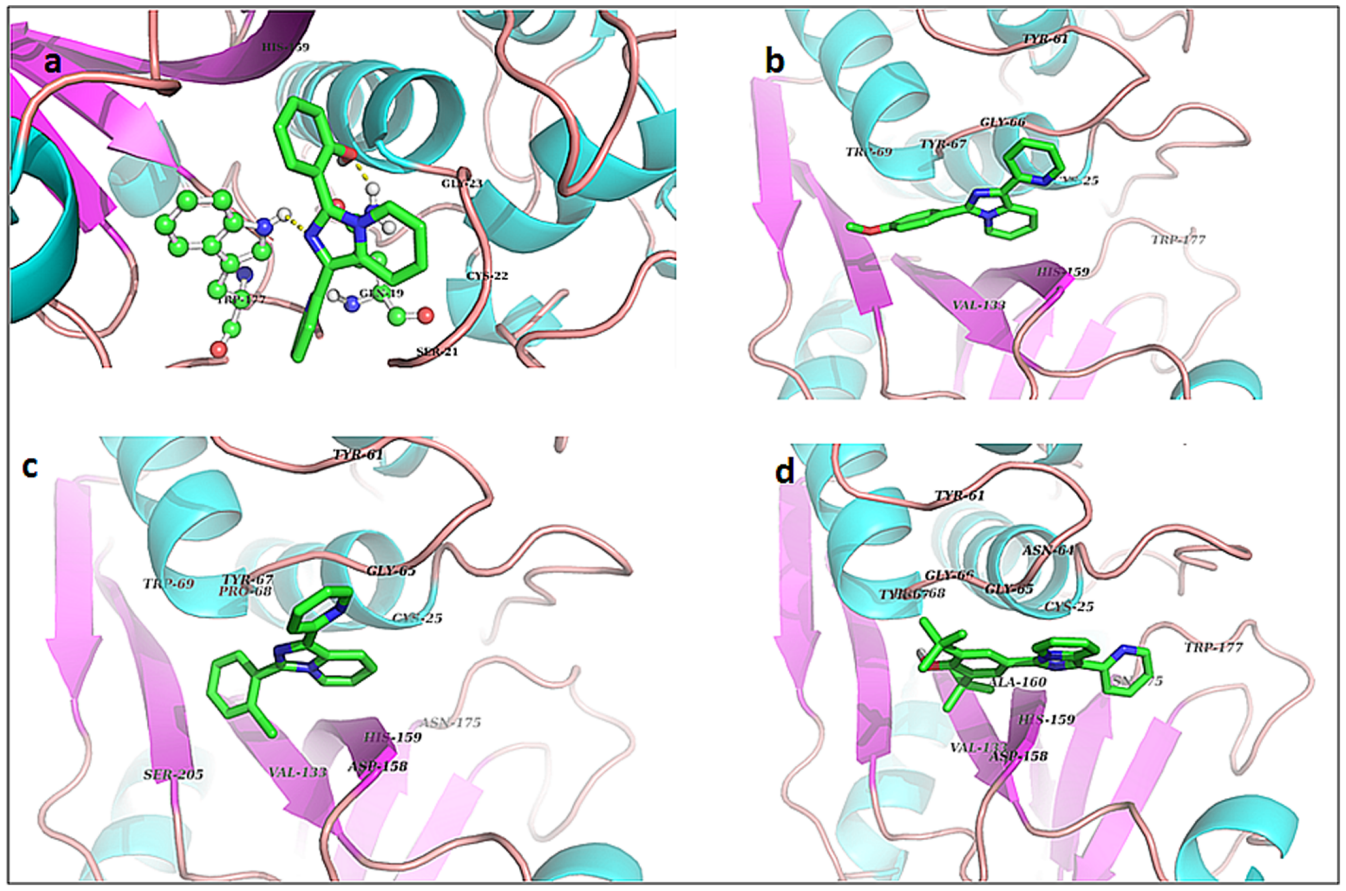
Interaction of compounds 3a (A), 3b (B), 3c (C) and 3d (D) within the active site of papain.

The findings of the above study have shown that the molecular interactions between the compounds **3a–d** and papain were very similar to the interactions observed for E-64c, a derivative of naturally occurring epoxide inhibitor (E-64c) **(** **Figure 1** **)** of cysteine proteases [31], [32], with papain; especially with regard to the hydrogen bonding and hydrophobic interactions of the ligands with conserved residues in the catalytic binding site (**Figure 2** **A–D**). Several papain residues participated in hydrophobic interactions with compounds **3a–d**, including Gln19, Cys25, Gly66 and Asp158. The pyridine moieties of compounds **3a–d** interact with S2 site of papain which includes (Tyr61, Asn64, Gly65 & Tyr67) amino acids (**Figure 2** **A–D**). The active site residues that were found to be key player in the interaction of compounds within the active site (mostly through hydrophobic interactions) were Cys25, Tyr61, His159 and Trp177, while Trp177, Gln19 were found to me making hydrogen bonds only with compound **3a**. Besides this many other residues were also found to be actively involved (**Table 1**). Furthermore, the binding energies for the compound **3a**, **3b**, **3c** and **3d** with papain were found to be −6.12, −5.76, −6.84 and −5.62 Kcal/mol respectively, which were in great agreement with our wet lab experiments; shall be discussed later (**Table 1**). This confirmed the accuracy of our docking protocol. Since, the binding energy is a direct measure of strength of interaction and our compounds **3a–d** showed stronger binding within the active site of papain in comparison to the inhibitor E-64c (ΔG: −4.04 Kcal/mol), therefore, the results suggest that these 1-substituted pyridylimidazo[1,5-*a*]pyridine derivatives (**3a–d**) could be potent inhibitors of papain like cysteine proteases.

**Table 1:** Molecular interaction studies of papain (PDB: 1PE6) with 1-substituted pyridylimidazo[1,5-*a*]pyridine derivatives.

Compounds	Binding energy (Kcal/mol)	Dock energy(Kcal/mol)	ComputationalK_d_ (µM)	Residues Involved	
				Hydrogen Bonding	Hydrophobic Interaction
3a	−6.12	−7.32	32.48	W177, Q19	Q19, S21, C22, G23, D158, H159, W177
3b	−5.76	−7.04	59.89	No Hydrogen Bond	C25, Y61, G66, Y67, W69, V133, H159, A160
3c	−6.84	−8.57	9.64	No Hydrogen Bond	C25, Y61, G65, Y67, P68, W69, V133, D158, H159, S205
3d	−5.62	−7.03	75.48	No Hydrogen Bond	C25, Y61, N64, G65, G66, Y67, P68, V133, D158, H159, A160, W177

**Figure 3:**
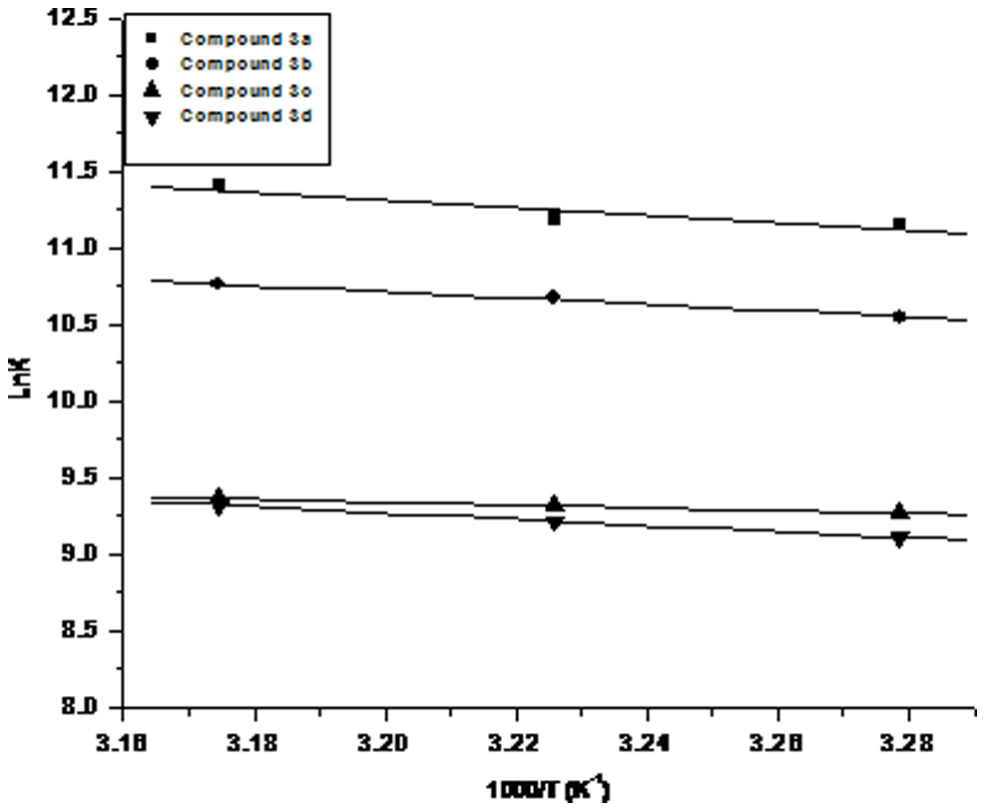
Van’t Hoff Plot of the effect of temperature on the inhibition constant of papain against 1-pyridylimidazo[1,5-*a*]pyridines derivatives.

The *in silico* interaction of compounds **3a–d** with papain, which were observed as discussed above, was validated with wet lab thermodynamics studies which showed that free energy of the binding (ΔG) of **3a**, **3b**, **3c** and **3d** with papain were −6.86, −6.55, −5.71 and −5.64 Kcal/mol, respectively at 37°C and the values were negative at all temperatures (32°C, 37°C and 42°C) studied, suggesting a spontaneous binding (**Table 2**). Interestingly, the observed *in silico* binding energies for the compounds **3a–d** against papain were found to be in great agreement (standard error ±2 Kcal/mol) with the value of free energy of binding (ΔG) observed during thermodynamics studies (**Table 1** **and** **2**). Similarly, enthalpy change (ΔH) of the binding was negative whereas entropy (ΔS) change of the binding was positive which indicated the exothermic and entropically driven nature of binding. This pattern of temperature dependence is characteristic of hydrophobic interaction [33]. As discussed earlier that all the compounds (**3a–d**) were found to interact with the active site residues of papain through hydrophobic interactions at most instances during *in silico* studies, the same was observed by the analysis of Van’t Hoff plots for all the proposed inhibitors at three different temperatures (32°C, 37°C and 42°C) in wet lab experiments (**Figure 3**). This proves the importance of these types of interactions in the positioning of compounds within the active site. Hence, thermodynamics as well as *in silico* study reveals that hydrophobic interactions favor binding of these proposed inhibitors with papain like cysteine proteases. Further wet lab results proposed the non competitive interaction of compounds (**3a**, **3c** & **3d**) with papain except for compound **3b** which showed competitive interaction. In sum up, the above results of molecular docking studies and thermodynamic analysis of compounds **3a–d** with papain showed that these compounds have the potential to be novel and unique cysteine protease inhibitors.

**Table 2:** Inhibition constant and thermodynamic parameter for inhibition of papain against 1-substituted pyridylimidazo[1,5-*a*]pyridine derivatives at different temperatures.

Ki (µM) Temperature						
Compound	32°C	37°C	42°C	aΔG (Kcalmol^−1^)	aΔH (KJmol^−1^)	aΔS (Jmol-K^−1^)
3a	14.3(±0.71)	13.7 (±0.57)	11.1(±0.52)	−6.86(±0.30)	−6.2(±0.88)	72.92 (±2.33)
3b	26.2(±1.10)	23.2(±1.09)	21.1(±0.92)	−6.55(±0.33)	−5.3(±0.80)	71.4 (±2.46)
3c	94.1(±4.42)	90.0(±4.50)	85.6(±3.59)	−5.71(±0.27)	2.3 (±0.31)	69.88 (±2.65)
3d	110.2(±4.85)	99.3(±4.36)	89.2(±3.74)	−5.64(±0.24)	−5.2 (±0.84)	59.73(±1.93)

a Value at 37°C.

(±) standard deviation; n = 3.

In the current study, the cysteine protease inhibitory activity of synthesized derivatives of 1-substituted pyridylimidazo[1,5-*a*] pyridine (**3a–d**)) was also performed against papain and the inhibition constants (K_i_) for the above said enzyme were observed to be 13.70, 23.20, 90.00 and 99.30 µM for compounds **3a**, **3b**, **3c** and **3d** respectively (**Table 3**). Furthermore, the calculated IC_50_ values were also found to be 13.40, 21.17, 94.50 and 96.50 µM for compounds **3a**, **3b**, **3c** and **3d** respectively (**Table 3**). Except compound 3b, rest of the compounds showed non competitive, reversible inhibitions but all the compounds irrespective of types of binding, showed hydrophobic and entropically driven interaction. These derivatives (**3a–j**) were eventually evaluated for their antibacterial activities against seven clinically important microbes (*S. aureus, P. vulgaris, Group D Streptococci*, *Bacillus sp.*, *E. coli*, *P. aeruginosa* and *S. morganii*). Here, we are showing the data of only four compounds (**3a–d**) because of their significant results (**Table 4**). All the compounds strictly followed the pattern of antiprotease activity towards bacterial growth except *P. vulgaris* and *E. coli* at one instance each **(** **Table 4** **)**. Since compound 3c & 3d do not have much difference in their IC50 values (**3c**-94.5 µM and **3d**-96.5 µM) against cysteine protease, papain and hence in antibacterial activity in all instances except one. It might be random due to so close in IC50 values. Compounds **3c** & **3d** are having much difference in their IC50 values (**3b**-21.17 µM and **3c**-94.5 µM) and they showed exact pattern for their antibacterial activity for all microbes except for *E. coli* at one instance. Although, *E. coli* does contain six major cysteine proteases but none belong to the CA clan of papain. It is argued that these compounds also inhibited the cysteine proteases of other clan than papain but with low efficacy.

**Table 3:** Name, Structure, IC50 & K_i_ of 1-substituted pyridylimidazo[1,5-*a*]pyridine derivatives against cysteine protease papain.

Compound	Type of inhibition	Ki (µM)	IC_50_ (µM)
2-(1-(pyridin-2-yl-imidazo[1,5-a]pyridin-3-yl)-phenol **(3a)**	Non- Competitive	13.7	13.4
3-(4-methoxy-phenyl)-1-pyridin-2-yl-imidazo[1,5-a]pyridine **(3b)**	Competitive	23.2	21.17
3-(2-chloro-phenyl)-1-pyridin-2-yl-imidazo[1,5-a]pyridine **(3c)**	Non -Competitive	90.0	94.5
2,6-di-*tert*-butyl-4-(1-pyridin-2-yl-imidazo[1,5-a]pyridin-3-yl)-phenol **(3d)**	Non -Competitive	99.3	96.5

**Table 4:** Antimicrobial activity of 1-substituted pyridylimidazo[1,5-*a*]pyridine derivatives.

Microorganism^a^								
Compound		1	2	3	4	5	6	7
**3a**	b **MIC_50_**	0.9(±0.04)	0.8(±0.04)	0.7(±0.03)	0.6(±0.03)	0.6(±0.03)	0.6(±0.03)	0.8(±0.04)
	b **MIC_90_**	1.7(±0.08)	1.6(±.08)	1.3(±0.06)	1.2(±0.06)	0.99(±0.04)	1.09(±0.05)	1.5(±0.09)
	b **MBC_50_**	1.8(±0.09)	1.6(±0.08)	1.3(±0.06)	1.2(±0.06)	1.3(±0.06)	1.1(±0.05)	1.6(±0.08)
	b **MBC_90_**	3.5(±0.17)	3.1(±0.15)	2.5(±0.12)	2.3(±0.11)	2.1(±0.07)	1.9(±0.09)	3.1(±0.16)
**3b**	**MIC_50_**	1(±0.05)	0.8(±0.04)	**NA**	0.9(±0.05)	1(±0.05)	1(±0.05)	**NA**
	**MIC_90_**	1.9(±0.1)	1.5(±.08)	**NA**	1.7(±0.08)	1.8(±0.06)	1.8(±0.09)	**NA**
	**MBC_50_**	2.3(±0.11)	1.6(±0.08)	**NA**	1.7(±0.08)	1.9(±0.09)	2.1(±0.1)	**NA**
	**MBC_90_**	4.5(±0.21)	3.2(±0.17)	**NA**	3.2(±0.15)	2.9(±0.11)	4.0(±0.17)	**NA**
**3c**	**MIC_50_**	1.2(±0.06)	1(±0.05)	0.9(±0.04)	0.9(±0.05)	0.6(±0.03)	1.2(±0.06)	1(±0.05)
	**MIC_90_**	2.4(±0.13)	1.9(±0.1)	1.7(±0.07)	1.7(±0.08)	1.1(±0.06)	2.2(±0.11)	1.9(±0.09)
	**MBC_50_**	2.4(±0.12)	2.2(±0.11)	1.8(±0.09)	1.9(±0.09)	1.2(±0.06)	2.4(±0.12)	2.1(±0.1)
	**MBC_90_**	4.7(±0.23)	4.3(±0.2)	3.4(±0.16)	3.8(±0.19)	2.3(±0.15)	4(±0.2)	4.1(±0.2)
**3d**	**MIC_50_**	1.2(±0.06)	0.9(±0.05)	1(±0.05)	1.4(±0.07)	1.2(±0.06)	1.2(±0.06)	1.1(±0.05)
	**MIC_90_**	2.3(±0.15)	1.7(±0.1)	1.9(±0.09)	2.6(±0.11)	2.3(±0.11)	2(±0.1)	2.1(±0.1)
	**MBC_50_**	2.6(±0.13)	1.7(±0.08)	2.3(±0.11)	2.5(±0.12)	2.5(±0.12)	2.4(±0.12)	2.4(±0.12)
	**MBC_90_**	5.2(±0.27)	3.4(±0.12)	4.4(±0.19)	4.7(±0.23)	4.9(±0.24)	4.4(±0.22)	4.4(±0.22)
**Ceftriaxone**	**MIC_50_**	3.8(±0.20)	0.4(±0.02)	0.08(±0.004)	0.8(±0.04)	0.07(±0.003)	1.5(±0.08)	0.9(±0.04)
	**MIC_90_**	6(±0.30)	0.8(±0.03)	1.4(±0.05)	1.6(±0.08)	0.12(±0.005)	3(±0.14)	1.5(±0.07)
	**MBC_50_**	7(±0.33)	0.9(±0.04)	1.5(±0.07)	1.7(±0.07)	0.14(±0.005)	3.2(±0.16)	1.7(±0.08)
	**MBC_90_**	10(±0.42)	1.2(±0.05)	1.9(±0.09)	3.1(±0.14)	0.24(±0.012)	5.5(±0.23)	3.3(±0.13)
**Clotrimazole**	**MIC_50_**	0.0009(±0.000045)	0.00084 (±0.000042)	**NT**	**NT**	45(±2.25)	40(±2.0)	**NT**
	**MIC_90_**	0.0016(±0.00008)	0.00145 (±0.000072)	**NT**	**NT**	85(±4.25)	75(±3.75)	**NT**
	**MBC_50_**	0.0014 (±0.00007)	0.0014 (±0.00007)	**NT**	**NT**	75(±3.75)	68(±3.4)	**NT**
	**MBC_90_**	0.003 (±0.00015)	0.0032 (±0.00016)	**NT**	**NT**	144(±7.2)	134(±6.7)	**NT**

a 1. *S. aureus*, 2. *P. vulgaris*, 3. *Group D Streptococci*, 4. Bacillus sp. 5. *E. coli*, 6. *Pseudomonas aeruginosa*, 7. *Serratia morganii* NA- Not active.

NT- Not tested.

(±) standard deviation; n = 3.

b Values in µg/ml.

Since, pyridylimidazo[1,5-*a*]pyridine derivatives is absolutely new scaffold towards antibacterial agents and hence, not any standard compound(s) of same scaffold is available for reference. So, Clotrimazole (1-[(2-chlorophenyl)(diphenyl)methyl]-1*H*-imidazole), an imidazole derivatives and Ceftriaxone (third-generation cephalosporin antibiotic with broad spectrum activity against Gram-positive and Gram-negative bacteria) have been used as positive control whereas DMSO has been used as negative control. All the above mentioned bacterial species have been shown to secrete certain cysteine proteases which play very important role in the pathogenecity of different diseases caused by these microorganisms. The minimum inhibitory concentration (MICs) of compounds (**3a–d**) (**Table 4**) against all tested bacteria except *E. coli* and *P. vulgaris*, were observed to be in great agreement with their respective inhibition constant (K_i_)/IC_50_ values against papain **(** **Table 3** **)** which clearly indicates that these compounds have the potential to inhibit the papain like cysteine proteases of these pathogens. The partition coefficient (logP) is a well-established measure of the compound’s lipophilicity. The distribution of calculated logP (cLogP) values of a majority of drugs in the market is in the range of zero to five. All the compounds studied except **3d**, showed good agreement with the criteria laid down for the prediction of a compound to be a potential drug (**Table 5**). All the compounds do not show any threat against toxicity risk assessment except compound **3d** which showed threat as tumorogenic effect due to the presence of isobutyl group. Among all the tested compounds, compound **3a** was the most potent whose MIC was the lowest among all the tested compounds and showed maximum drug score and positive values for drug likeness.

**Table 5:** Prediction of antibacterial compounds as drugs (http://www.organic-chemistry.org).

Compound	acLogP prediction	bsolubility prediction	cMw	dDrug Likeness prediction	eOverall Drug Likeness Score	^f^M	gT	hIE	iRE	H-bond accaeptor	H-bond donor	No. of rotational bonds
3a	3.84 (4.13) [3.2]	−5.16 (−4.11)	287	0.71	0.41	N	N	N	N	4	1	2
3b	4.04 (4.05) [4.19]	−5.48 (−4.64)	301	−0.23	0.41	N	N	N	N	4	0	3
3c	4.75 (4.58) [4.64]	−6.19 (−5.23)	305	0.16	0.16	N	N	N	N	3	0	2
3d	6.88 (6.57) [7.15]	−7.48 (−5.72)	399	−16.3	0.09	N	Y	N	Y	4	1	4

a compounds to have a reasonable propability of being well absorbt their logP value must not be greater than 5.0.

b more than 80% of the drugs on the market have a (estimated) logS value greater than −4.

c more than 80% of all traded drugs have a molecular weight below 450.

d 80% of the drugs have a positive druglikeness value.

e drug score combines drug likeness, cLogP, logS, molecular weight and toxicity risks^ f^Mutagenicity.

g Tumorigenicity.

h Irritant effect.

i Reproductive effect.

**() (**Tetko et al, 2005).

**[]** Chemdraw.

In summary, the results of the present study have established that 1-substituted pyridylimidazo[1,5-*a*]pyridine derivatives could be candidate for novel and potent inhibitors of papain like cysteine proteases, which play deleterious role in the progression of different diseases caused by diverse microorganisms. Therefore, this group of compounds could be the subject of future research to confront the challenges with resistant microorganisms that is a major threat globally.

## Supporting Information

File S1 **Types of inhibitions with Ki (Compounds 3a–3d).**
(DOC)
