# Supplementary material for: Design, Synthesis, Evaluation and Thermodynamics of 1-Substituted Pyridylimidazo[1,5-a]Pyridine Derivatives as Cysteine Protease Inhibitors
Source: PLoS One. 2013 Aug 5;8(8):e69982. doi: 10.1371/journal.pone.0069982 (PMC3734177; doi:10.1371/journal.pone.0069982)
Supplement: File S1 — Types of inhibitions with Ki (Compounds 3a–3d). (DOC) [file pone.0069982.s001.doc]

**Fig S: Types of inhibitions with Ki (Compounds 3a-3d)**

**Fig S1: Showing Dixon and Line Weaver Burk Plot for compound 3a**

**Fig S2: Showing Dixon and Line Weaver Burk Plot for compound 3b**

-120

-100

-80

-60

-40

-20

0

20

40

60

80

100

120

140

160

0

2

4

6

8

10

**4mM**

**5mM**

Ki=90.0uM

**1/[V]**

**[I]uM**

**Fig S3: Showing Dixon and Line Weaver Burk Plot for compound 3c**

**Fig S4: Showing Dixon and Line Weaver Burk Plot for compound 3d**
